# Theragnostic Radionuclide Pairs for Prostate Cancer Management: ^64^Cu/^67^Cu, Can Be a Budding Hot Duo

**DOI:** 10.3390/biomedicines10112787

**Published:** 2022-11-02

**Authors:** Anupriya Chhabra, Mathew L. Thakur

**Affiliations:** 1 Department of Radiology, Thomas Jefferson University, Philadelphia, PA 19107, USA; 2 Department of Radiation Oncology, Thomas Jefferson University, Philadelphia, PA 19107, USA; 3 Department of Urology, Thomas Jefferson University, Philadelphia, PA 19107, USA; 4 Sidney Kimmel Cancer Center, Thomas Jefferson University, Philadelphia, PA 19107, USA

**Keywords:** prostate cancer, theragnostic, theragnostic radionuclide pairs, radionuclide therapy, bone metastases, ^64^Cu/^67^Cu

## Abstract

Prostate cancer (PCa) is one of the preeminent causes of mortality in men worldwide. Theragnostic, a combination of therapy and diagnostic, using radionuclide pairs to diagnose and treat disease, has been shown to be a promising approach for combating PCa. In PCa patients, bone is one of the most common sites of metastases, and about 90% of patients develop bone metastases. This review focuses on (i) clinically translated theragnostic radionuclide pairs for the management of PCa, (ii) radionuclide therapy of bone metastases in PCa, and (iii) a special emphasis on emerging theragnostic radionuclide pair, Copper-64/Copper-67 (^64^Cu/^67^Cu) for managing the disease.

## 1. Introduction

Prostate cancer (PCa) is the most commonly diagnosed male malignancy worldwide [1]. Reportedly, PCa is more prevalent in the developed world, where it is a leading cause of cancer mortality among Swedish men and the second leading cause of cancer death in the United Kingdom [2]. In the rest of Europe, it is the third most commonly diagnosed cancer [3]. It is estimated that in 2022, in the United States, newly diagnosed PCa will account for 14% of all new cancer cases, and the estimated deaths will account for 5.7% of all cancer deaths [4].

**Figure 1:**
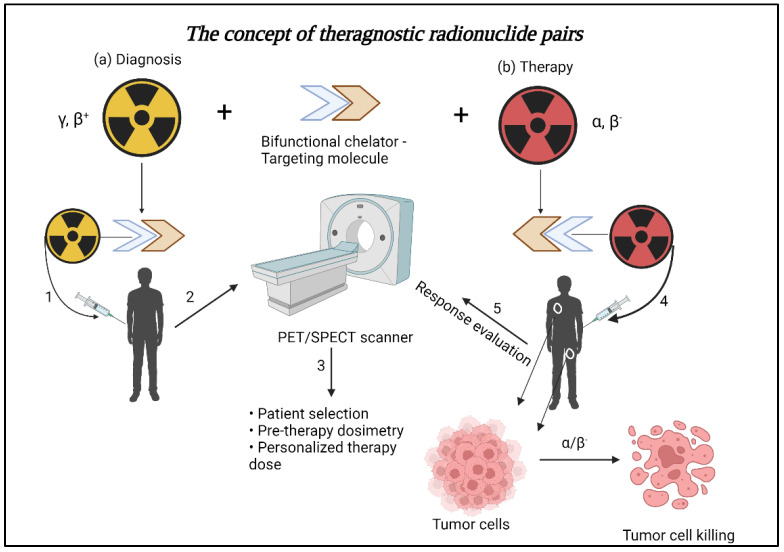
The schematic shows the concept of theragnostic pairs. Targeting molecule conjugated with a bifunctional chelator (BFC) and radiolabeled with a diagnostic radionuclide (gamma/positron (γ/β^+^) emitting) is injected into the patient (1). Molecular imaging is performed using positron emission tomography (PET) or single photon emission computed tomography (SPECT) to identify lesions (2). Patient selection is made based on scan findings, and pre−therapy dosimetry is performed to calculate personalized therapy dose. (3). For therapy, the same BFC−targeting molecule tagged with a therapeutic radionuclide is injected into the patient (4). Alpha/beta particles (α/β^−^) cause the tumor cell killing, and response evaluation is done with molecular imaging (5). Created with BioRender.com, accessed on 25 September 2022.

A testosterone-reducing drug combined with androgen deprivation therapy is the first-line therapy for metastatic PCa. Most patients respond to these regimens initially. The disease, however, advances to metastatic castration-resistant prostate cancer (mCRPC) [5]. At this stage, patient survival may be improved by using theragnostic radionuclide pairs [6]. Theragnostic radionuclide pairs are a combination of diagnostic and therapeutic radionuclides sharing the same target inside the body. They can belong to the same or different elements and can have similar chemistry and pharmacokinetics. The use of theragnostic radionuclide pairs can be highly beneficial in the management of cancer as well as other life-threatening diseases. First, a diagnostic radiopharmaceutical is administered to the patient, and molecular imaging is performed. The findings allow to select a patient who is most likely to benefit from the therapy. Second, the biodistribution of the diagnostic radiopharmaceutical further helps to determine the possible adverse effects of therapeutic radiopharmaceutical. Third, the location of the primary lesion and its tumor volume determined from the pre-therapy scan, the estimated highest whole-body dose, and critical tissue radiation dose help to calculate a personalized therapeutic dose. Fourth, post-therapy diagnostic radiopharmaceutical help in predicting response to the treatment [7,8,9] (Figure 1).

A number of theragnostic radionuclide pairs have been translated clinically, such as Iodine-123/Iodine-131 (^123^I/^131^I), Gallium-68/Lutetium-177/Actinium-225 (^68^Ga/^177^Lu/^225^Ac), Technetium-99m/Rhenium-188 (^99m^Tc/^188^Re), ^99m^Tc/Yttrium-90 (^99m^Tc/^90^Y) etc. [10,11]. Radioiodine was the first theragnostic agent used in medicine to treat thyroid abnormalities. ^123^I/^131^I is an effective and true theragnostic radionuclide pair as both are chemically identical [12]. In thyroid conditions, ^123^I-sodium iodide (NaI) is used to determine the percentage uptake in the thyroid. Following resection of differentiated thyroid carcinomas, ^123^I-NaI identifies residual functional tissue in the thyroid bed and evaluates the amount of metastases and iodide avidity [13]. The therapeutic activity of ^131^I-NaI is personalized based on the size of the patient’s gland and the percentage of iodide uptake in order to deliver a 100–150 Gy radiation dose to the thyroid [14].

^68^Ga-prostate specific membrane antigen-11 (^68^Ga-PSMA-11) has been successfully used in mCRPC patients for primary staging, lesion detection in biochemical recurrence cases, and assessing response to therapy with ^177^Lu-/^225^Ac-PSMA-617 [15]. Bone is one of the most common sites of metastases in PCa patients as approximately 90% of mCRPC patients develop bone metastases. Bone lesions can cause pain, disability, and skeletal events, as well as deteriorates quality of life [16]. This review discusses the theragnostic radionuclide pairs for PCa management and radionuclide therapy of bone metastases in patients (Table 1).

Amongst the PCa theragnostics, Copper-64 and Copper-67 (^64^Cu/^67^Cu) is an emerging theragnostic radionuclide pair. Their nuclear properties are highly suitable for diagnosis and therapy. The half-life (t½) of ^64^Cu is 12.7 h, long enough for uptake and biodistribution studies of a radiopharmaceutical, including antibodies that may require a longer time for localization. Copper-64 is a positron-emitting radionuclide (E_β_^+^ = 0.653 MeV) that permits positron emission tomography (PET) imaging and has beta emissions (E_β_^−^_max_ = 0.579 MeV), which may be useful for theragnostic applications. Copper-67, on the other hand, decays with beta emissions of (E_β_^−^_max_ = 0.561 MeV) with t½ of 2.57 d, long enough to cause tumor cell killing but short enough not to induce excessive radiation burden to normal tissues. The coordination chemistry of copper is such that the radionuclides can be linked with several chelators, peptides, antibodies, and small molecules [17]. Since both belong to the same element and share similar chemistry, ^67^Cu can be used in lieu of ^64^Cu for radionuclide therapy. This paper briefly discusses the production, and clinical applications of ^64^Cu/^67^Cu in PCa patients.

## 2. Peptide Receptor Radionuclide Therapy (PRRT)

Increased prostate-specific antigen (PSA) levels are one of the hallmarks of PCa. Expression of prostate-specific membrane antigen (PSMA) is positively linked with PSA levels. PSMA is a type 2 integral membrane glycoprotein with intracellular, transmembrane, and extracellular domains. It consists of 750 amino acids and two monomers [18]. PSMA is expressed at low levels in the cytoplasm of normal prostate epithelial cells. In salivary glands, lacrimal glands, kidneys, and gastrointestinal tract it is overexpressed physiologically [19]. However, in 90–95% of PCa cells, it dwells in the luminal epithelium of prostatic ducts where it is up to one thousand times more abundant [20]. Moreover, PSMA expression levels are highest in metastatic and castrate-resistant diseases. This, differential expression in normal tissues and cancer cells, as well as its ability to be internalized after ligand binding, make PSMA an appealing target for theragnostics [20,21]. For targeting PCa, PSMA has been radiolabeled with theragnostic radionuclide pair ^68^Ga and ^177^Lu. ^68^Ga is a generator-produced positron (1.92 MeV) emitting radionuclide having a t_1/2_ of 68 min. Like ^68^Ga, ^177^Lu is also a trivalent cation with a t_1/2_ of 6.7 d. Having E_β_^−^_max_ = 0.496 MeV for therapy and E_γ_ = 0.208 MeV (10.4%) for imaging ^177^Lu renders itself as an attractive theragnostic radionuclide [22].

### 2.1. Gallium-68-Prostate Specific Membrane Antigen-11 (^68^Ga-PSMA-11) (Locametz)

^68^Ga-PSMA-11 belongs to a class of urea-based, peptidomimetic PSMA inhibitors. It is indicated for imaging PSMA-positive lesions in men with PCa. Diagnostic imaging is performed with PET in those patients who have (i) suspected metastases, (ii) are candidates for definitive therapy, and (iii) with suspected recurrence based on elevated serum PSA levels [23]. The sensitivity of ^68^Ga-PSMA-11 correlates positively with serum PSA levels and performs relatively well at low PSA levels. A clinical trial in PCa patients reported a sensitivity of 47% and specificity of 90% [24].

### 2.2. Lutetium-177-Prostate Specific Membrane Antigen-617 (^177^Lu-PSMA-617) (Pluvicto)

The PSMA-binding therapeutic radioligand ^177^Lu-PSMA-617 (^177^Lu-vipivotide tetraxetan) is indicated for the treatment of PSMA-positive adult mCRPC patients, who have previously been treated for androgen receptor pathway inhibition and with taxane-based chemotherapy (Table 2). A ^68^Ga-PSMA-11 pre-therapy scan is performed to determine PSMA expression in PCa. Those who express PSMA, qualify for 6 cycles of intravenous administration of 7.4 GBq ^177^Lu-PSMA-617 every 6 weeks. The radiopharmaceutical is distributed in various organs within 2.5 h of injection and 60–70% of which binds to human plasma proteins [9]. ^177^Lu-PSMA-617 beta emissions cause DNA damage, resulting in cytotoxicity in PSMA-expressing and nearby cells [25]. An international, open-label, phase 3 trial of ^177^Lu-PSMA-617 reported imaging-based progression-free survival (PFS) and overall survival of 8.7 and 15.3 months. Patients reported fatigue, dry mouth, nausea, anemia, back pain, arthralgia, decreased appetite, constipation, and diarrhea [26]. The highest mean calculated absorbed dose for lacrimal gland was 92 Gy for the cumulative administered activity of 44.4 GBq (6 × 7.4 GBq). For salivary gland the dose was 28 Gy, for the large intestine it was 92 Gy, for kidneys 19 Gy, for urinary bladder 14 Gy and for blood marrow 1.5 Gy [27]. The primary route of excretion is the kidneys, with a mean terminal elimination half-life of 41.6 h. Patients with kidney impairment may be more susceptible to the renal toxicity. A follow-up patient study found a link between cumulative radiation doses and worsening kidney function after 13 ± 9 months of therapy [28]. However, in another radioligand therapy study, did not induce deterioration in kidney function as observed in a subgroup of mCRPC patients having kidney impairment [29].

### 2.3. Lutetium-177-J591 (^177^Lu-J591)

Anti-PSMA monoclonal antibody J591 has been used for targeting mCRPC. A dose of 1665 MBq/m^2^ given at a 2-week interval showed a median survival of 42.3 months and a reduction in PSA levels. 79.6% of the patients had positive ^68^Ga-PSMA-11 imaging. Patients with low PSMA expression had poor responses. At high doses, PSA levels decreased with an increase in overall survival as well as increased toxicity [30]. The authors added that the cumulative radiation dose was higher when ^177^Lu-J591 was administered in fractions.

### 2.4. Actinium-225-Prostate Specific Membrane Antigen-617 (^225^Ac-PSMA-617)

**Figure 2:**
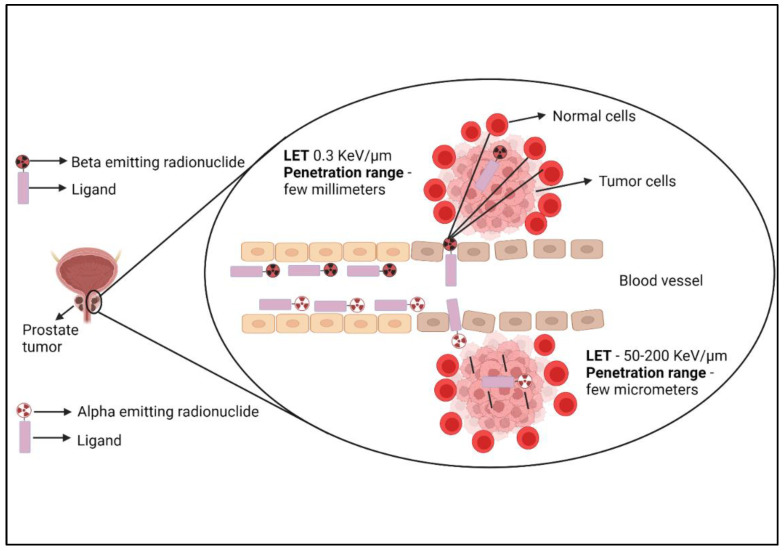
Figure shows tumor cell killing by therapeutic radiopharmaceutical. Beta particles have linear energy transfer (LET) of 0.3 KeV/µm and a penetration range of a few millimeters and may cause minor damage to nearby normal cells. Alpha emitting radionuclides have high therapeutic efficiency (LET-50–200 KeV/µm) and penetration range of few micrometers and cause little or no effect on nearby normal cells.

Alpha emitting radionuclides are evaluated for PRRT due to their short penetration range (50–80 μm) and high linear energy transfer (80–100 keV/μm). The alpha radiations induce double-strand DNA breaks, and DNA cluster breaks causing highly effective cell killing (Figure 2). ^225^Ac is an alpha-emitting radionuclide (6 MeV) with a t_1/2_ of 9.9 d. A study with ^225^Ac-PSMA-617 treatment (100 KBq/kg body weight) showed a ≥90% decline in PSA levels in 82.3% of PCa patients. Furthermore, 88.2% of patients had a more than 50% decline in lesions avidity, as showed in ^68^Ga-PSMA-PET/CT scan (Table 2). In 64.7% of patients, all the metastatic lesions were completely resolved. There were side effects in the form of xerostomia, bone marrow toxicity, and renal impairment [31,32].

**Table 2:** Clinical utility and activity of therapeutic radiopharmaceuticals for prostate cancer and bone metastases [25,31,33,34,35,36,37].

	Radiopharmaceutical	Trade Name	Indication	Dose	Outcome
1	Lutetium-177- Prostate specific membrane antigen (PSMA)-617	Pluvicto	Treatment of PSMA-positive adult metastatic Castration resistant prostate cancer patients (mCRPC), previously treated for androgen receptor pathway inhibition and with taxane-based chemotherapy	6 cycles of 7.4 GBq every 6 weeks	Progression-free survival—8.7 months, Overall survival (OS)—15.3 months
2	Actinium-225-PSMA-617	-	PSMA expressing mCRPC patients	100 KBq/kg body weight	Decline in prosate specific antigen level and lesion avidity for Gallium-68-PSMA-11 positron emission tomography/computed tomography
3	Radium-223 chloride	Xofigo	Treatment of CRPC patients and symptomatic bone metastases, and no known visceral metastatic disease	6 cycles, 50 kBq per kg body weight, every 4-week	OS-14.9 months, improved quality of life
4	Strontium-89 chloride	Metastron	Patients with painful bone metastases lesions	148 MBq	Pain relief and improved quality of life
5	Samarium-153-ethylenediaminetetramethylenephosphonic acid	Quadramet	Painful metastatic bone lesions	37 MBq/kg	Pain relief

## 3. Radionuclide Therapy for Bone Metastases

Metastatic PCa cells interact with bone microenvironments and release various growth factors. These growth factors disturb the tightly regulated intercellular communication between osteoblasts and osteoclasts and dysregulate their activity. It results in a profusion of new, disordered bone. In turn, osteoblasts produce growth factors that stimulate the growth and survival of PCa cells. This bidirectional positive-feedback loop gives rise to osteoblastic bone metastases, characteristic of PCa, accounting for significant morbidity, bone fracture, pain, and even death [36]. Bone-seeking therapeutic radiopharmaceuticals, having an affinity for hydroxyapatite, have been used for the treatment of bone metastases.

### 3.1. Radium-223-Chloride (^223^RaCl_2_) (Xofigo)

^223^RaCl_2_ (t_1/2_-11.4 d) is an alpha-emitting radiopharmaceutical indicated for treating CRPC patients with symptomatic bone metastases but no known visceral metastatic disease. A dose of 50 kBq/kg body weight is administered every 4-week intervals for 6 cycles. Six injections of ^223^RaCl_2_ per patient have shown overall median survival of 14.9 months, improved quality of life, favorable safety profiles, and low rates of myelosuppression. Moreover, the development of skeletal complications was delayed, and the risks of spinal cord compression were significantly reduced. A 3-year follow-up analysis suggested long-term safety of ^223^RaCl_2_ as no association was found between ^223^RaCl_2_ treatment and secondary malignancies [33,38].

### 3.2. Strontium-89-Chloride (^89^SrCl_2_) (Metastron)

Strontium-89 is a beta-emitting radionuclide (E_β_^−^_max_ = 1.463 MeV) with a t_1/2_ of 50.5 d. Strontium-89 chloride acts as a calcium analog and is retained in metastatic bone lesions for much longer period than in normal bone. The recommended dose, which clears rapidly from the blood, is 148 MBq. Clinical trials have witnessed relief of pain which usually begins 10–20 d after its administration and lasts up to 6 months, improving the quality of life [34,39].

### 3.3. Samarium-153-Ethylenediaminetetramethylenephosphonic Acid (^153^Sm-EDTMP) (Quadramet)

^153^Sm has a t_1/2_ of 46.3 h and emits medium-energy beta-particles and gamma-photons. A dose of 37 MBq/kg body weight is administered intravenously [35]. ^153^Sm-EDTMP is effective in treating bone metastases due to its fast blood clearance, high bone uptake, and low nonosseous uptake. Patients often decrease their analgesics. Turner et al. reported pain alleviation in 65% of patients over 4 to 35 weeks, and in some patients, transitory myelosuppression and delayed thrombocytopenia were observed. Sartor et al. reported that repeat dosing in patients (37 MBq/kg body weight) having painful bone metastases was safe and effective. The treatment is reasonable in patients who still have bone pain after an initial dose and have adequate hematologic function [40,41,42,43,44].

### 3.4. Gallium-68/Lutetium-177 [(bis(Phosphonomethyl)Carbamoyl] Methyl-7,10-bis(Carboxymethyl)-1,4,7,10 Tetraazacyclododec-1-Yl) Acetic Acid (^68^Ga/^177^Lu-BPAMD)

This has been used in PCa patients with widespread and painful skeletal metastases. A high tumor dose is delivered owing to the long half-life of the ^177^Lu (6.7 d), which results in a considerable decrease in the osteoblastic activity of the bone metastases, as observed in a subsequent PET/CT using ^68^Ga-BPAMD [45,46].

### 3.5. Technetium-99m Methyl Diphosphonate/Hydroxy Methylene Diphosphonate/Rhenium-188 Hydroxyethylidine Diphosphonate (^99m^Tc-MDP/HMDP/^188^Re-HEDP)

^99m^Tc (t_1/2_ = 6 h, E_γ_ = 0.140 MeV) and ^188^Re (t_1/2_ = 16.9 h, E_γ_ = 0.155 MeV (15%), E_β_^−^_max_ = 2.1 MeV) are transition elements of group VII b of the periodic table and share similar chemistry. MDP/HMDP and HEDP are molecules with a strong affinity toward hydroxyapatite present in the actively growing bone. ^99m^Tc-MDP/HMDP and ^188^Re-HEDP are used for diagnosing bone lesions and their palliative therapy, respectively. It is reported that multiple injections of ^188^Re-HEDP increase the response rate and duration of pain relief and improve the quality of life with moderate toxic effects [47,48,49].

## 4. Copper-64 and Copper-67

Copper is the third most abundant transition trace metal in humans, after iron and zinc (Zn) [50]. Copper plays a vital role in the metabolic pathways of cells, such as in neurotransmitter synthesis and pigment formation. Copper is a transition metal with a mass number 63.55 and an atomic number 29. With electronic configuration [Ar] 3d^10^4s^1^, Copper belongs to group 11 of the periodic table. Copper has multiple oxidation states; Cu(II) is the most favorable for radiopharmaceutical preparation. Cu(II) is less labile to ligand exchange because of crystal field stabilizing energy. It can exist in chloride, nitrate, bromide, etc., forms and is generally stable and soluble in water. Copper has two naturally occurring and stable isotopes, of which 69.15% is ^63^Cu, and 30.85% is ^65^Cu. It has 27 radioactive isotopes and out of these ^60^Cu (E_β_^+^ = 2.9 MeV, t_1/2_ = 23.7 min), ^61^Cu (E_β_^+^ = 1.21 MeV, t_1/2_ = 3.33 h), ^62^Cu (E_β_^+^ = 2.9 MeV, t_1/2_ = 9.7 min), ^64^Cu (E_β_^+^ = 0.653 MeV, E_β_^−^_max_ = 0.57 MeV, t_1/2_ = 12.7 h) and ^67^Cu (E_β_^−^_max_ = 0.561 MeV, t_1/2_ = 2.57 d) are used for imaging and radiotherapy [51,52].

### 4.1. Copper-64

**Figure 3:**
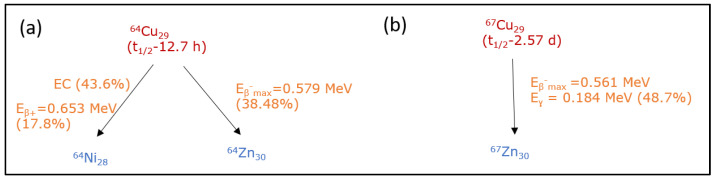
Decay scheme of (**a**) Copper-64 (**b**) Copper-67.

Amongst all the radioactive isotopes of copper, ^64^Cu is the most studied owing to its ideal nuclear properties. Copper-64 decays to Nickel-64 (^64^Ni) with E_β_^+^ = 0.653 MeV (17.8%) and electron capture (43.6%). It also decays to Zinc-64 (^64^Zn) with E_β_^−^_max_ = 0.579 MeV (38.48%) (Figure 3a). It has a t_1/2_ of 12.7 h, which is suitable for imaging using small as well as large molecules such as antibodies and peptides. The relatively short t_1/2_ of ^64^Cu does not add unnecessary radiation burden to the patient after imaging studies have been performed, and yet, ^64^Cu radiopharmaceuticals can be shipped long distances without excessive radioactivity decay [53].

### 4.2. Production of Copper-64

^64^Cu can be produced by neutron bombardment in a reactor or by proton bombardment in a cyclotron. To produce ^64^Cu in a reactor, ^63^Cu(n,γ)^64^Cu or ^64^Zn(n,p)^64^Cu nuclear reactions are used. The use of ^63^Cu as the target, produce ^64^Cu with low specific activity. ^64^Zn target requires fast neutron flux and produces ^65^Zn (t_1/2_ = 245 d) as an impurity. These factors limit the availability of ^64^Cu from the reactor. Cyclotron production involves ^64^Ni(p,n)^64^Cu reaction, which is widely used in the US to meet the need for ^64^Cu. In this production method, enriched nickel (99.6%) is electroplated onto a gold disk or on a copper substrate having a gold layer and bombarded with protons to obtain ^64^Cu. It has been reported that bombarding 40 mg Ni with 15.5 MeV protons for 4 h at 60 µA gives 18.5 GBq ^64^Cu. After production, copper is carefully separated from nickel by an ion-exchange column chromatography [54,55].

### 4.3. Copper-64 Radiopharmaceuticals for Imaging Prostate Cancer

For radiolabeling of biomolecules with ^64^Cu, a bifunctional chelator (BFC) is needed. A suitable BFC should have rapid radiolabeling kinetics, and the radiolabeled complex should be stable in vivo and in vitro. Most commonly used BFC for ^64^Cu are acyclic ligands diacetyl-bis-N-4-methylthiosemicarbazone (ATSM), pyruvaldehyde bis(N4-methylthiosemicarbazonato (PTSM), polyazamacrocyclic chelators 1,4,8,11-Tetraazacyclotetradecane-1,4,8,11-tetraacetic acid (TETA) and 2,2′,2″,2‴-(1,4,7,10-Tetraazacyclododecane-1,4,7,10-tetrayl)tetraacetic acid (DOTA), 1,4,7-triazacyclononane,1-glutaric acid-4,7-acetic acid (NODAGA), triazacyclononanes 2,2′,2″-(1,4,7-triazacyclononane-1,4,7-triyl)triacetic acid (NOTA), bicyclic tetraazamacrocycles (cross-bridged analogues of DOTA and TETA), hexaazamacrocycles (sarcophagines), bispidine and N_2_S_2_ type diaminedithiol ligands.

#### 4.3.1. Copper-64-Chloride (^64^CuCl_2_)

Ionic ^64^CuCl_2_ has also been used to screen patients with PCa. 185–370 MBq ^64^CuCl_2_ administered had given good tumor to background ratio. It was shown that the tracer delivered a few nanograms of copper to cells and had no known cytotoxic effect. The radiotracer had a rapid blood clearance. Dosimetry studies in healthy volunteers suggested the liver, intestine, and pancreas as the critical organs [56].

#### 4.3.2. Copper-64-Diacetyl-bis-N-4-Methylthiosemicarbazone (^64^Cu-ATSM)

In most tumor microenvironments, increased cell proliferation and decreased neoangiogenesis lead to low oxygen levels. In many cases, the hypoxic conditions render tumors resistant to chemotherapy and radiation therapy. ^64^Cu-ATSM has been employed in these cases as a diagnostic marker for hypoxia imaging to assess the prognosis. In ^64^Cu-ATSM, copper is present in a ^64^Cu(II) oxidation state. It is considered that inside the tumor, it gets reduced to ^64^Cu(I), the complex becomes unstable, and ^64^Cu is trapped within the tumor. ^64^Cu-ATSM has no urinary excretion and can also image PCa [57,58,59,60].

#### 4.3.3. Copper-64-Prostate Specific Membrane Antigen-617 (^64^Cu-PSMA-617)

Grubmuller et al. examined the diagnostic potential of ^64^Cu-PSMA-617 in 29 PCa patients. The preliminary results demonstrated a high potential of ^64^Cu-PSMA-617 for PET/CT imaging in patients with recurrent disease and in selected patients with locally advanced disease. The images displayed very high lesion-to-background ratio with excellent resolution of the detected lesions [61]. Using ^64^Cu-NODAGA-PSMA another study examined 23 PCa patients with the recurring disease and a few individuals with advanced local disease. The lesions detected in the prostate, lymph nodes, and distant metastases sites were significantly associated with PSA values. ^64^Cu-NODAGA-PSMA uptake considerably increased between 30 min and 1–3 h post-injection. The authors concluded that ^64^Cu-NODAGA-PSMA PET is stable in vivo and is a promising imaging tool [62].

#### 4.3.4. Copper-64-Sarcophagine-bisPSMA (^64^Cu-SAR-bisPSMA)

Clinical trials are ongoing with ^64^Cu-SAR-bisPSMA to identify PSMA-expressing mCRPC. Patient’s recruitment started in 2021 and is currently recruiting [63]. In the phase I study one administration of 200 MBq ^64^Cu-SAR-bisPSMA will be given to the patients for dosimetric determination, two administrations for dose escalation studies, and three for cohort expansion study. The purpose of the research is to determine the safety and efficacy of ^67^Cu-SAR-bisPSMA in patients.

#### 4.3.5. Copper-64-TP3805 (^64^Cu-TP3805)

Thakur et al. developed and evaluated TP3805, a peptide analog of pituitary adenylate cyclase-activating peptide (PACAP) having a high affinity for VPAC1 receptors. VPAC1 receptors (a combined for vasoactive intestinal peptide (VIP) and PACAP) are minimally expressed in normal cells and benign tumors but expressed in high density on many types of malignant cells. TP3805 was conjugated with N_2_S_2_ (diaminedithiol(N_2_S_2_-Benzoyl)_2_) chelating agent at the C-terminus and radiolabeled with ^64^Cu. A kit was also formulated to make it convenient for reliable and routine radiolabeling [64].

In a clinical study of 25 PCa patients, PET imaging with ^64^Cu-TP3805 identified lesions in the prostate gland as confirmed by post-surgical histology. Digital autoradiography (DAR) with ^64^Cu-TP3805 identified 98% PCa foci, 100% high-grade intraepithelial neoplasia, and other malignant lesions. For benign lesions, DAR was negative. The study demonstrated that ^64^Cu-TP3805 is highly specific for PCa and merits further investigation [65]. Another study with ^64^Cu-TP3805 in urothelial bladder cancer patients (n = 19) clearly visualized the lesions due to negligible urinary excretion [66]. ^64^Cu-TP3805 can also identify other malignant lesions and bone metastases. In a clinical study of 19 breast cancer patients [67], positron emission mammography uptake value/background value ratios of the 15 min, post-injection images did not alter significantly for up to 5 h of imaging indicating high in vivo stability of ^64^Cu-TP3805.

Hence, it was proposed that ^67^Cu-TP3805, with excellent theragnostic qualities (Table 3 and Table 4), can be used for PRRT in PCa patients. A few advantages of ^67^Cu-TP3805 over existing PRRT ^177^Lu-PSMA-617 are:

(i) ^177^Lu-PSMA-617 requires a pre-therapy scan with ^68^Ga-PSMA-11. On the other hand, VPAC receptors are expressed in all PCa patients, eliminating the need for a patient qualifying pre-therapy scan.
(ii) ^177^Lu-PSMA-617 therapy may requires amino acid and botulinum toxin pretreatment. However, ^67^Cu-TP3805 has no uptake in salivary glands. Hence, can treat patients without undergoing multiple pretreatment procedures.
(iii) ^67^Cu-TP3805 has no urinary excretion and can treat bladder cancer, primary PCa their metastatic lesions as well as involved lymph nodes.
(iv) Cancer stem cells express VPAC receptors [68,69] and can be targeted with ^67^Cu-TP3805, perhaps preventing recurrence of the disease.

**Table 4:** Comparing theragnostic characteristics of ^67^Cu-TP3805 with ^177^Lu-PSMA.

Characteristics	^177^Lu-PSMA	^67^Cu-TP3805	Advantages of ^67^Cu-TP3805
Tissue range (mm)	0.6	0.6	Same as ^177^Lu
Receptor expression on prostate cancer (PCa)	80–85%	100%	No patient screening procedure required 100% of the PCa patients can be treated
Tissue distribution			
Salivary glands	Yes	No	No xerostomia No botulinum toxin pretreatment required
Renal	Yes (cortex and medulla)	Cortex only	No renal damage No amino acid treatment required
Bladder	Yes	No	Primary PCa lesion can be diagnosed and treated
Metastatic lesions	Yes	Yes	All distant metastatic lesions can be treated
Cancer stem cells	No	Yes [69,70]	Cancer stem cells can be targeted Minimize recurrence

### 4.4. Production of Copper-67

Copper-67 decays to ^67^Zn with E_β_^−^_max_ = 0.561 MeV, E_γ_ = 0.184 MeV (48.7%) and a t_1/2_ of 2.57 d (Figure 3b) [17]. Although ^67^Cu can be produced from the reactor, the low yield and expensive target material (^67^Zn) limit the availability of reactor-produced ^67^Cu. In a cyclotron, ^67^Cu can be produced using Zn, Ni, Cu, and Ga as target material (Table 5). The main route of production using Zn as the target material is a ^68^Zn(p,2p)^67^Cu reaction with proton-beam energy 38–50 MeV. Enriched ^68^Zn targets not only enhance ^67^Cu yield but also reduce co-production of other Cu-radionuclides. At the proton beams of up to 30 MeV, the ^70^Zn(p,α)^67^Cu reaction is feasible without the co-production of ^64^Cu. The reaction requires enriched ^70^Zn (95.47%), which is expensive and makes recovery and re-use of the irradiated target material a vital task. Using deuteron beams of 7–25 MeV ^70^Zn(d,x)^67^Cu reaction seems promising also [70]. Alpha beams are used to irradiate nickel in a ^64^Ni(α,p)^67^Cu reaction, but the reaction has a low yield. Production of ^67^Cu using ^65^Cu(α,2p)^67^Cu reaction yields ^67^Cu with a very low specific activity. Production of ^67^Cu from Ga using ^71^Ga(p,x)^67^Cu nuclear reaction requires 20–40 MeV proton beam energy and 24 h irradiation time. The disadvantages of the reaction are low yield and high ^64^Cu contamination. Furthermore, the low melting point of gallium makes target preparation difficult for nuclear bombardment [71,72,73,74,75,76,77].

Photonuclear production using ^68^Zn(γ,p)^67^Cu reaction is another method for ^67^Cu production. It is reported that bombarding 55.5 g enriched ^68^Zn with 40 MeV bremsstrahlung photons in electron linear accelerator for 53.5 h gives 62.9 GBq activity and >1850 Bq/mg ^67^Cu at the end of bombardment (EOB) without detecting ^64^Cu as a contaminant. The enriched target material is mandatory to avoid the co-production of zinc and copper radionuclides as impurities [78].

### 4.5. Copper-67 Radiopharmaceutical for Prostate Cancer Therapy

#### Copper-67-Sarcophagine-bis-Prostate Specific Membrane Antigen (^67^Cu-SAR-bisPSMA)

Clinical trials are being conducted to investigate the safety and efficacy of ^67^Cu-SAR-bisPSMA in PSMA-expressing mCRPC patients [63]. Patients will receive two doses at the recommended dose level determined by dose escalation during the cohort expansion phase.

## 5. Conclusions

PCa theragnostics involve various radiopharmaceuticals that target both primary lesions as well as bone metastases. FDA approved, ^177^Lu-PSMA-617 increases OS and imaging-based PFS in PCa patients. In mCRPC patients, targeted alpha therapy (TAT) with ^225^Ac-PSMA-617 causes relatively low toxicity; hence, it is an effective and safe treatment option. However, clinical trials are required to compare the therapeutic effects and survival benefits with existing clinical treatments. TAT of bone metastases in PCa with ^223^RaCl_2_ remains an important component of the treatment paradigm as it has proven survival benefits. Tumor-specific TAT seems promising due to its high therapeutic efficiency, minimal damage to normal tissue, and ability to target small volume disease. ^89^SrCl_2_ and ^153^Sm-EDTMP enable pain relief and improve the quality of life of PCa patients having bone metastases.

^64^Cu and ^67^Cu are chemically identical radionuclides. Both have similar in vivo behavior which facilitates the use of the former as a predictor of the biodistribution and toxicity of the latter. Moreover, a ^64^Cu pre-therapy dosimetry scan helps calculate a personalized ^67^Cu patient therapy dose. ^67^Cu-bisPSMA and ^67^Cu-TP3805 are emerging radiopharmaceuticals for PCa theragnostics. The efficient and optimized production methods yielding large amounts of ^64^Cu and ^67^Cu with high specific activity can enhance routine availability and enable its more widespread use.

## Figures and Tables

**Table 1:** Half-life and energies of theragnostic duos and therapeutic radionuclides.

S.No	Radionuclide	Half-Life	Energies in MeV			
			Alpha	Beta (Max)	Gamma	Positron
1	^68^Ga	68 min	-	-	-	1.92
	^177^Lu	6.7 d	-	0.496	0.208	-
	^225^Ac	9.9 d	6	-	-	-
2	^99m^Tc	6.0 h	-	-	0.140	-
	^188^Re	16.9 h	-	2.1	0.155	-
3	^64^Cu	12.7 h	-	0.579	-	0.653
	^67^Cu	2.57 d	-	0.561	0.184	-
4	^223^Ra	11.4 d	6	-	-	-
5	^89^Sr	50.5 d	-	1.463	-	-
6	^153^Sm	46.3 h	-	0.807	-	-

**Table 3:** Comparison of characteristics of radionuclide ^67^Cu and ^177^Lu.

	^67^Cu	^177^Lu
Half-life (d)	2.6	6.7
Beta tissue range (mm)	0.6	0.6
Energies (MeV)	E_β_^−^_max_—0.561 E_γ_—0.184	E_β_^−^_max_—0.496 E_γ_—0.208
Hospitalization required	No	No
Production method	Accelerator	Reactor

**Table 5:** Nuclear reactions for Copper-67 production and yields [70].

Nuclear Reaction	Energy Range (MeV)	^67^Cu (MBq/µA)	
		End of Bombardment	After 72 h Cooling Time
^68^Zn(p,2p)^67^Cu	50–38	166	74
^70^Zn(p,α)^67^Cu	24–8	113	50.6
^70^Zn(d,x)^67^Cu	25–7	123	54.9
^64^Ni(α,p)^67^Cu	33–9	18.9	8.4
^71^Ga(p,x)^67^Cu	40–20	12.5	5.6

Note: Data given in the table for the nuclear reactions are reported with enriched target and 24 h irradiation time.
